# Biomanufacturing and Curcumin-Loading of Human Choroid Plexus Organoid-Derived Extracellular Vesicles from a Vertical-Wheel Bioreactor to Alleviate Neuro-Inflammation

**DOI:** 10.3390/biomedicines13051069

**Published:** 2025-04-28

**Authors:** Justice Ene, Laureana Muok, Vanessa Gonzalez, Nicolas Sanchez, Aakash Nathani, Falak Syed, Zixiang Leonardo Liu, Mandip Singh, Tristan Driscoll, Yan Li

**Affiliations:** 1 Department of Chemical and Biomedical Engineering, FAMU-FSU College of Engineering, Florida State University, Tallahassee, FL 32310, USA; je17d@fsu.edu (J.E.); laureana1.muok@famu.edu (L.M.); vgonzalez2@fsu.edu (V.G.); ns20d@fsu.edu (N.S.); fs23i@fsu.edu (F.S.); leo.liu@eng.famu.fsu.edu (Z.L.L.); tdriscoll2@eng.famu.fsu.edu (T.D.); 2 College of Pharmacy and Pharmaceutical Sciences, Florida A&M University, Tallahassee, FL 32310, USA; akash.nathani96@gmail.com (A.N.); mandip.sachdeva@famu.edu (M.S.)

**Keywords:** human pluripotent stem cells, choroid plexus organoids, extracellular vesicles, Vertical-Wheel bioreactors, lyophilization, drug loading

## Abstract

**Background:** Choroid plexus is a complex structure in the human brain that is responsible for the secretion of extracellular vesicles (EVs) in cerebrospinal fluid. Few studies to date have generated choroid plexus (ChP) organoids differentiated from human induced pluripotent stem cells (hiPSCs) and analyzed their secreted EVs. The scalable Vertical-Wheel bioreactors (VWBRs) provide low shear stress and a controlled environment. **Methods:** This study utilized VWBRs for the differentiation of hiPSCs into ChP organoids and generation of the secreted EVs compared to a static culture. Additionally, this study loaded curcumin into ChP organoid-derived EVs, performed EV lyophilization, and determined the ability of the re-hydrated EVs to alleviate neuro-inflammation. **Results:** The results demonstrated that the VWBR culture exhibited more aerobic metabolism and active glucose and glutamine consumption than the static control. Consequently, the ChP markers and Endosomal Sorting Complexes Required for Transport-dependent and -independent EV biogenesis genes were significantly upregulated (2–3-fold) in the VWBR, producing four-fold-higher EVs per mL media than the static control. The EVs retained similar size and zeta potential after lyophilization and re-hydration. The cells exposed to amyloid beta 42 oligomers and treated with the curcumin-loaded re-hydrated EVs showed high viability and the reduced inflammatory response determined by TNF-α and IL-6 expression. **Conclusions:** This study demonstrates a scalable bioreactor system to promote ChP organoid differentiation and generation of EV-based cell-free therapeutics to treat neural inflammation in various neurological disorders.

## 1. Introduction

Cerebrospinal fluid (CSF) is secreted by the choroid plexus (ChP) [1,2], which is a mass of highly vascularized epithelial tissue that is located in the lateral, third, and fourth ventricles of the brain [3,4]. While various studies have revealed the ChP to be vital for normal brain development, central nervous system (CNS) homeostasis, and repair after disease and trauma, it is still considered as a relatively understudied tissue. The ChP also acts as a physical barrier that prevents toxic metabolites from entering the brain while working to remove moieties that bypass it. This barrier combines structural diffusion restraint from tight junctions between plexus epithelial cells and specific exchange mechanisms across the interface in order to control the internal CNS microenvironment [3]. The ChP is also a major contributor to the extracellular vesicles (EVs), the membrane-enclosed nanovesicles (50–1000 nm) released by cells through membrane budding or exocytosis, in CSF [5]. However, there are still many unanswered questions regarding the ChP–CSF system, such as how gene transcription and protein translation are regulated and the mechanisms of protein secretion that are utilized by the ChP [6].

Recently, it has been observed that ChP could be the location of serious complications related to aging, such as Alzheimer’s disease [3,4,7]. It is known that dysfunction of brain barriers contributes to neurological conditions; however, there is additional concern when the ChP is dysfunctional during neural degeneration. These observations further support the need for additional investigation of the ChP tissue. Obtaining a better understanding of the ChP could potentially hold the key to overcoming current barriers in drug delivery and the development of therapies for a wide range of neurological diseases. Current models that are used are primarily animal models or CSF from human volunteers. These models have not been able to provide information regarding the specific cellular and tissue sources of various secreted proteins [8].

To accomplish this, a human pluripotent stem cell (hPSC)-derived ChP organoid model was developed which can generate CSF [8]. It was observed that the organoids possessed similar morphological and functional features as human ChP. By applying small molecules such as dopamine, the organoids formed a tight barrier that mimicked the similar permeability to small molecules that has been observed in vivo [8]. The CSF components secreted by the ChP were determined, as well as the developmental factors and human-specific signaling proteins that may play key roles in brain development and homeostasis. Our previous study also generated ChP organoids from hPSCs in static culture-based differentiation and performed the preliminary EV secretion analysis [9]. Further studies are necessary to better understand the EVs that are secreted from these organoids and their therapeutic potential [10].

EVs have low immunogenicity, high stability in blood, and can directly deliver drugs to cells and are used as therapeutics [11]. Due to their unique ability in cell–cell communication, EVs are extremely beneficial for drug delivery. By loading them with exogenous drugs, EVs could change the functional state of recipient cells [12]. To date, the effects of EVs and their cargo have been studied on cancer, cardiovascular diseases, autoimmune diseases, inflammatory diseases, etc. [13]. Another major advantage of EVs is their ability to cross the blood brain barrier (BBB) [14,15], the blood–cerebrospinal fluid barrier (BCSFB) consisting of choroid plexus epithelial cells, and the arachnoid barrier. These barriers are highly selective as a major obstacle in the treatment of neurological inflammatory and degenerative disorders and diseases. By using EVs for drug delivery, a variety of therapeutic agents and biological cargos would be able to cross the BBB to be transported to the brain [14,16,17,18].

Neuroinflammation is a common symptom of aging and often caused by several components such as overexpression of pro-inflammatory factors by the microglia. An increase in the production of these factors can have detrimental effects in neurodegenerative diseases. Curcumin is a natural polyphenol sourced from turmeric and most reputable for its anti-inflammatory, antioxidant, and anticancer properties, which could be a key therapeutic for neurodegeneration [19,20,21,22]. Curcumin suppresses inflammation by blocking the nuclear transcription factor-kB that is responsible for regulating tumor necrosis factor (TNF)-α, a mediator for inflammation. Despite the benefits of curcumin, its clinical application has been hindered due to its low solubility and stability in vivo. A possible solution is to load curcumin into EVs. Various studies have shown that loading curcumin into EVs can be beneficial by improving its solubility, increasing its circulation time, preserving drug therapeutic activity, and improving delivery to brain [23]. The curcumin-loaded EVs alleviated IL-1β-induced catabolic effects by promoting cell viability and migration, reducing apoptosis and phosphorylation of Erk1/2, PI3K/Akt, and p38 MAPK, thereby modulating pro-inflammatory signaling pathways [24].

One of the greatest challenges for the use of stem cells and the secreted EVs in practical applications is the availability of culture systems for large-scale production. There is a growing interest in bioreactors as a possible solution, due to their ability to provide a controlled microenvironment [25,26]. While various bioreactors have successfully been used for the expansion of different types of stem cells, such as stirred tank bioreactors and perfusion bioreactors [26,27], there have been very few studies that have used bioreactors for the generation of organoid-derived EVs. One class of bioreactors that are showing great promise for both cell expansion and EV production are Vertical-Wheel bioreactors (VWBRs). VWBRs have shown promising results for the expansion of human induced pluripotent stem cells (hiPSCs) and human mesenchymal stem cells (hMSCs) on microcarriers and as aggregates. This is in part due to their unique design features, such as the vertical impeller and U-shaped bottom that result in gentle and uniform mixing and particle suspension with reduced power input and agitation speeds, as shown in our previous studies for EV production from bone marrow-derived hMSCs and hiPSCs [28,29,30,31]. Our previous studies also generated EVs from retinal organoids and natural killer cells in the VWBRs [32,33]. Despite these successes, few studies have examined their use in ChP organoid differentiation and ChP EV production.

This study investigated ChP organoid differentiation from hiPSCs and the EV secretion in a VWBR, with comparison to static cultures. The VWBR resulted in more aerobic metabolism and active glucose and glutamine consumption, demonstrating distinctly different metabolic pathways compared to the static control, consistent with our previous study [34]. Consequently, the ChP markers and Endosomal Sorting Complexes Required for Transport (ESCRT)-dependent and -independent EV biogenesis genes were significantly upregulated in the VWBR, producing four-fold-higher EVs per mL medium than the static control. Moreover, this study loaded curcumin into ChP organoid-derived EVs, performed EV lyophilization, and determined the ability of the re-hydrated EVs in alleviating neuro-inflammation. The EVs were successfully lyophilized and re-hydrated with similar size and zeta potential. The curcumin-loaded lyophilized EVs were tested on cells exposed to amyloid beta 42 oligomers. The cells showed high viability and the reduced inflammatory response with EV treatment as determined by *TNF-α* and *IL-6* expression. This study presents a novel approach to generate hiPSC-derived ChP organoids and the secreted EVs in VWBRs as well as designing EV-based cell-free therapeutics to treat neural inflammation in neurological disorders.

## 2. Materials and Methods

### 2.1. Undifferentiated hiPSC and hMSC Cultures

The human iPSK3 cell line was obtained by transfecting human foreskin fibroblasts (from a deidentified donor) with plasmid DNA encoding reprogramming factors octamer-binding transcription factor 4 (OCT4), NANOG, SRY-box transcription factor 2 (SOX2), and LIN28 (kindly provided by Dr. Stephen Duncan, Medical College of Wisconsin) [35,36]. The cells were cultured in mTeSR Plus serum-free medium (StemCell Technologies, Inc., Vancouver, BC, Canada) on a growth factor-reduced Matrigel-coated surface (BD Biosciences, Franklin Lakes, NJ, USA). Before seeding, one mL of 1% Matrigel was added to a 6-well tissue culture plate at 37 °C for at least one hour. The coating solution was then removed, and 1.5 × 10^6^ cells were seeded in 3 mL of fresh mTeSR^TM^ medium containing 10 µM Rho-kinase (ROCK) inhibitor Y-27632 (dilution, 1:1000, Sigma-Aldrich, St. Louis, MO, USA). After 24 h, Y-27632 was removed by feeding with fresh mTeSR^TM^ medium daily. The cells were ready to be passaged by Accutase (Life Technologies, Carlsbad, CA, USA) every 4–6 days.

Frozen adipose-derived hMSCs at passage 1 were acquired from the Tulane Center for Stem Cell Research and Regenerative Medicine. The adipose tissue-derived hMSCs were isolated from the subcutaneous abdominal adipose tissue from three de-identified healthy donors that were younger than 45 years old with a body mass index lower than 25. The adipose tissue-derived hMSCs were seeded (2500 cells/cm^2^) in Minimal Essential Medium (MEM) (Life Technologies, Carlsbad, CA, USA)-based media, with 10% fetal bovine serum (FBS) and 1% penicillin/streptomycin (Life Technologies). Regular media change was performed every 2–4 days. The cells were passaged or harvested with a 0.25% Trypsin/ethylenediaminetetraacetic acid (EDTA) (Invitrogen, Grand Island, NY, USA) solution. The FBS in the culture medium was switched to EV-depleted FBS prior to medium collection for EV isolation. The isolated EVs were used to optimize the EV loading process.

### 2.2. ChP Organoid Differentiation

Static ChP differentiation was performed using a modified protocol based on our previous study [17]. hiPSCs were seeded onto a 24-well ultra-low attachment (ULA) plate (Corning Incorporated, Corning, NY, USA) at 3 × 10^5^ cells/well, in 1 mL of fresh Dulbecco’s Modified Eagle Medium/Nutrient Mixture F-12 (DMEM/F12) plus 2% B27 serum-free supplement (cat. no. 17504044, Life Technologies under ThermoFisher, Carlsbad, CA, USA) and B27 serum-free medium containing 10 µM ROCK inhibitor Y-27632 (Sigma-Aldrich). On day 1, the medium was changed with DMEM/F12-B27 only to remove Y-27632. Then, the medium was changed every 2–3 days. Starting on day 10, 3 μM CHIR99021 (CHIR, a Wnt activator, Sigma-Aldrich) and 20 ng/mL bone morphogenetic protein (BMP)4 (Peprotech, Inc., Rocky Hill, New Jersey, USA) were added to the media every other day until day 15. Then, the medium was switched to DMEM/F12-B27 only. On day 24, the cells were harvested and the spent media were collected during day 16–24 for EV isolation. A small volume (1–3 mL) of spent media was also collected at each feeding for metabolite analysis.

### 2.3. ChP Organoid Derivation in Vertical-Wheel Bioreactors

Vertical-Wheel bioreactor-based ChP differentiation was performed similar to static differentiation in our previous publications [32,34]. The Vertical-Wheel bioreactors (0.1 L, PBS Biotech Inc., Camarillo, CA, USA) were seeded with hiPSCs at a density of 1 × 10^5^ cells/mL in 100 mL of fresh DMEM/F12-B27 medium containing 10 µM ROCK inhibitor Y-27632. The cells were then incubated at 37 °C for 24 h at 25 rpm to form small spheroids. On day 1, agitation speed was adjusted to 40 rpm and 50% of the medium was changed with DMEM/F12-B27. Going forward, 50% of the medium was changed every 2–3 days. Starting on day 10, 3 μM CHIR and 20 ng/mL BMP4 were added to the DMEM/F12-B27 media every other day until day 15. Then, the medium was switched to DMEM/F12-B27 only. On day 24, the cells were harvested for mRNA isolation. The spent media were collected during day 16–24 for EV isolation. A small volume (1–3 mL) of spent media was also collected at each feeding for metabolite analysis.

### 2.4. Image Analysis for the ChP Organoids

Following formation of the aggregates at day 1, images were taken every 2–3 days using a Nikon Eclipse Ti-U inverted microscope and attached DS-Qi1 monochrome digital camera (Nikon Corporation, Melville, NY, USA). Images were analyzed to calculate the parameters of aggregates, including perimeter, area, and roundness. Specifically, the captured images were converted to binary images using ImageJ software 1.54f (http://rsb.info.nih.gov/ij) and analyzed with the “particle analysis tool”. Through particle analysis, the perimeter and area of each aggregate in the images can be calculated, indicating the size of the aggregates (=5–10). The aggregate circularity was indicated by the roundness factor.

### 2.5. Metabolite Analysis

The supernatants (1–3 mL) were removed from the bioreactor and static cultures during ChP organoid differentiation and frozen for later metabolite analysis. The spent media were thawed and analyzed with a BioProfile Flex2 analyzer (Nova Biomedical, Waltham, MA, USA) for metabolite concentrations, including ions, glucose, lactate, glutamine, ammonia, etc. The mol/mol ratios of lactate production to glucose consumption and the ammonia production to glutamine consumption were calculated to reveal the metabolic pathways.

### 2.6. Reverse Transcription-Quantitative Polymerase Chain Reaction (RT-qPCR) Analysis

Total RNA was isolated from the cell samples using the RNeasy Mini Kit (Qiagen, Valencia, CA, USA) according to the manufacturer’s protocol, followed by treatment with the DNA-Free RNA Kit (Zymo, Irvine, CA, USA). Reverse transcription was carried out using 2 μg of total RNA, anchored oligo-dT primers, and Superscript III (Invitrogen, Carlsbad, CA, USA, according to the manufacturer’s protocol). The primers were designed using the software Primer-BLAST (NIH Database) (Supplementary Table S1). The gene β-actin was used as an endogenous control for the normalization of expression levels. RT-qPCR reactions were performed on an ABI7500 instrument (Applied Biosystems, Foster City, CA, USA), using SYBRI Green PCR Master Mix (Applied Biosystems). The amplification reactions were performed as follows: 2 min at 50 °C; 10 min at 95 °C; and 40 cycles of 95 °C for 15 s; 55 °C for 30 s; and 68 °C for 30 s. The Ct values of the target genes were normalized to the Ct values of the endogenous control β-actin. Fold changes in gene expression were calculated using the comparative Ct method $—2^{{-(∆C}_{t\;treatment}-{∆C}_{t\;control})}$—to obtain the relative expression levels.

### 2.7. Extracellular Vesicle Isolation

For EV isolation experiments, conditioned media were sequentially spun (500× *g* for 5 min, 2000× *g* for 10 min, 10,000× *g* for 30 min) to remove cell debris, apoptotic bodies, large vesicles, etc. Polyethylene glycol (PEG)-6000 was added to the supernatant to a final concentration of 8% (*w*/*v*) PEG in 0.5 M NaCl and stored for 24 h at 4 °C in order to enrich EVs, as previously demonstrated [37,38,39]. The solution was spun at 3000× *g* for one hour and supernatant was discarded. The remaining pellet was suspended in 1 mL phosphate-buffered saline (PBS) and ultracentrifuged at 120,000× *g* for 70 min at 4 °C to remove residual PEG. The EV pellet was then re-suspended in 200 µL PBS using a benchtop shaker at 1500 rpm for 5 min. EVs were diluted to 10^8^–10^9^ particles per mL in PBS for nanoparticle tracking analysis [37,39].

### 2.8. Nanoparticle Tracking Analysis (NTA)

Particle-size distribution and zeta potential of isolated EVs were assessed using a NanoSight LM10-HS instrument (NTA 3.4 Build 3.4.003, Malvern Instruments, Malvern, UK) configured with a blue (488 nm) laser and sCMOS camera and a ZetaView instrument (ZetaView^®^ TWIN PMX-220, Particle Metrix Inc, Mebane, NC, USA), which utilizes the dynamic light scattering (DLS) technique at 25 °C with a 90° scattering angle. ZetaView Analysis software was used for data processing as previously explained. All the EV samples were prepared by diluting with PBS (particle-free) in a 1:1000 ratio [40,41]. For NanoSight measurements, for each replicate, three videos of 60 s were acquired with camera shutter speed fixed at 30.00 ms. To ensure accurate and consistent detection of small particles, the camera level was set to 13, and the detection threshold was maintained at three. The laser chamber was cleaned thoroughly with particle-free water between each sample reading. The collected videos were analyzed using NTA3.0 software to obtain the mode and mean size distribution, as well as the concentration of particles per mL of solution. Compared to the mean size, the mode size is usually a more accurate representation because the vesicle aggregates may affect the mean size.

### 2.9. Transmission Electron Microscopy (TEM)

TEM was performed to confirm the morphology of EVs. Briefly, EV isolates were resuspended in 50–100 μL of sterile filtered PBS. For each sample, intact EVs (15 µL) were dropped onto Parafilm. A carbon-coated 400 Hex Mesh Copper grid (Electron Microscopy Sciences, EMS, Hatfield, PA, USA) was positioned using forceps with the coating side down on top of each drop for one hour. Grids were washed with sterile filtered PBS three times and then the EV samples were fixed for 10 min in 2% paraformaldehyde (EMS, EM Grade). After washing, the grids were transferred on top of a 20 µL drop of 2.5% glutaraldehyde (EMS, EM Grade) and incubated for 10 min. Grid samples were stained for 10 min with 2% uranyl acetate (EMS Grade). Then, the samples were embedded for 10 min with 0.13% methyl cellulose and 0.4% uranyl acetate. The coated sides of the grids were left to dry before imaging on the HT7800 transmission electron microscope (Hitachi America, Ltd., Santa Clara, CA USA).

### 2.10. Western Blot for Exosomal Markers

EV and cell samples were lysed in radio-immunoprecipitation assay (RIPA) buffer (150 mM sodium chloride, 1.0% Triton X-100, 0.5% sodium deoxycholate, 0.1% sodium dodecyl sulfate, 50 mM Tris, pH8, and 1X Thermo Scientific™ Halt™ Protease Inhibitor Cocktail) (Thermo Fisher Scientific Inc., Waltham, MA, USA). Samples were incubated for 20 min on ice and spun down at 14,000 rpm for 20 min. The cleared supernatants were collected, and a Bradford assay was carried out to determine protein concentration. Protein lysate concentration was normalized based on the Bradford assay and denatured at 100 °C in 2 x Laemmli Sample buffer for 5 min. Equal amounts of protein sample (3–10 μg) were loaded into each well. Proteins were separated by 12% Bis-Tris-SDS gels and transferred onto a nitrocellulose membrane (Bio-Rad, Hercules, CA, USA). For the detection of non-phosphorylated proteins, the non-specific protein binding to membranes was blocked by incubation in 1% *w*/*v* non-fat dry milk in Tris-buffered saline (10 mM Tris-HCl, pH 7.5, and 150 mM NaCl) with 0.1% Tween 20 (*v*/*v*) (TBST) for one hour. Membranes were incubated overnight in the presence of the primary antibodies (Supplementary Table S2) diluted in blocking buffer at 4 °C. Afterward, the membranes were washed four times with TBST and then incubated with a horseradish peroxidase (HRP)-conjugated secondary antibody at 1:5000 for one hour. The blots were washed and imaged on a Biorad ChemiDoc Imaging System (Bio-Rad, Hercules, CA, USA).

### 2.11. Loading EVs Using Sonication, Incubation, and Freeze–Thaw Methods

A curcumin solution was prepared by dissolving curcumin (Sigma-Aldrich) in dimethyl sulfoxide (DMSO, Sigma-Aldrich) to a final concentration of 10 mg/mL. Prior to sonication, 450 µL of isolated EV suspension was mixed with 50 µL of curcumin solution. After vortexing for 30 s, the solution was placed in the sonicator (Fisher Scientific, Hampton, NH, USA) that was set to 2 watts. The curcumin–EV solution was sonicated for 5 cycles with 2 s of sonication at 2–5 s of rest between each cycle. This cycling procedure was repeated twice. The curcumin–EV solution was then incubated for one hour at 37 °C. After incubation, 250 µL of 24% (*w*/*v*) PEG was added to the 500 µL curcumin–EV solution. After incubation at 4 °C for 24 h, the solution was spun at 3000× *g* for one hour and supernatants were collected for determining the loading efficiency. The remaining pellet was suspended in 1 mL PBS and ultracentrifuged at 10,000× *g* for 30 min at 4 °C. The EV pellet was then resuspended in 200 µL PBS.

For loading via incubation and freeze–thaw, 100 µL of isolated EVs were mixed with 50 µL of curcumin stock solution. PBS was then added to achieve a final volume of 500 µL. The solution was vortexed for 30 s and then incubated at 37 °C for 24 h. After incubation, 250 µL of 24% (*w*/*v*) PEG was added to the 500 µL curcumin–EV solution for 24 h at 4 °C and unloaded curcumin was removed as previously described. For the freeze–thaw method, 100 µL of EV solution was mixed with 50 µL of curcumin stock solution. PBS was then added to achieve a final volume of 500 µL and the solution was vortexed for 30 s. The solution then underwent 2–3 cycles of incubation for 15 min at room temperature and 15 min at −80 °C. An amount of 250 µL of 24% (*w*/*v*) PEG was then added to the 500 µL curcumin–EV solution for 24 h at 4 °C and unloaded curcumin was removed as previously described.

Loading efficiency was calculated by measuring the concentration of the supernatant (S1), which was collected after centrifugation to remove unloaded curcumin. The concentration of S1 was determined using a standard linear curve. This curve was derived using a microplate reader (BioTek Instruments, Inc., Santa Clara, CA, USA) to obtain the values of 100%, 50%, 25%, 12.5%, 6.25%, and 0% samples of curcumin solutions that were diluted with PBS. The following equation was used to calculate the loading efficiency of curcumin in EVs.

$$=\left|\frac{\left(Concentration\;of100\%\;Curcumin\;Solution-Concentration\;of\;S1\right)}{\left(Concentration\;of\;100\%\;Curcumin\;Solution\right)}\right|\times 100$$

### 2.12. EV Lyophilization and Re-Hydration

EV lyophilization was carried out using a Labconco Dry Ice Benchtop Freeze Dryer (Labconco, Kansas City, MO, USA). A 150–200 μL solution of EV samples was lyophilized for 24 h in the presence or absence of trehalose (1:1 ratio, Sigma-Alderich). The protein amounts of the EVs were measured by the Bradford assay (Bio-Rad), which was used to determine the amount of trehalose to be used. Trehalose at a 50 mM in solution was added at a 1:1 weight ratio to the proteins within the EVs. The temperature was set to −80 °C and the vacuum was set to 0.5 mbar. The resulting lyophilized EV samples can be stored at room temperature for long-term use (one week to three months). For functional testing, lyophilized EVs were re-hydrated in 100 µL of PBS in the presence or absence of trehalose. After washing, the EVs were resuspended in PBS and used for NTA and cell culture experiments.

### 2.13. EV Effects on Amyloid Beta (Aβ) 42 Oligomer-Stimulated Cells

Aβ42 oligomers are known to induce a proinflammatory response. It has also been proposed that the accumulation of Aβ42 oligomers in the brain is involved in the pathogenesis of Alzheimer’s disease [42,43]. To prepare oligomers of Aβ (1–42) peptide, biotinylated Aβ (1–42) (Bachem, Torrance, CA, USA) was fully dissolved at 0.5 mg/mL in hexafluor-2-propanole (HFIP, Sigma-Alderich) [44,45]. An amount of 10 μL of HFIP Aβ (1–42) solution was dispensed into a siliconized Snap-Cap microtube, put in a desiccator to completely evaporate HFIP, and thereafter stored at −80 °C. Oligomer solutions were prepared freshly for each experiment. The stock was dissolved in 10 μL of DMSO (to 105 μM) and incubated for 3 h at room temperature. Oligomers of Aβ (1–42) were added in 24-well plates at 1 μM to the neural progenitor cells for viability testing and ChP organoid cultures derived from hiPSCs for neuro-inflammation testing. After Aβ treatments for two days, the cells were exposed to the loaded fresh EVs (1 × 10^9^ EVs per well), loaded and lyophilized EVs (1 × 10^9^ EVs per well), unloaded fresh EVs (1 × 10^9^ EVs per well), or nothing. The cells were then harvested for mRNA isolation and evaluation of inflammation markers.

### 2.14. Live/Dead Assay

The cells were evaluated for viability using the Live/Dead^TM^ staining kit (Molecular Probes, Eugene, OR, USA) according to the manufacturer’s protocol. After treating with EVs, the spheroids were trypsinized, washed with PBS, and then incubated in DMEM-F12 containing 3–10 μM calcein-AM (green) and 8 μM ethidium homodimer I (red) for 20 min at room temperature and protected from light. The images were acquired by a fluorescence microscope (Zeiss Axio Observer, Wixom, MI, USA). For some experiments, the samples after staining were acquired with a BD FACSCanto™ II flow cytometer (Becton Dickinson, Franklin Lakes, NJ, USA) and analyzed using FlowJo software (v10.10).

### 2.15. Statistical Analysis

Experimental results were expressed as means ± standard deviation (SD). Statistical comparisons were performed by one-way ANOVA and Tukey’s post hoc test for multiple comparisons, and significance was accepted at *p* < 0.05. For comparisons of two conditions, Student’s *t*-test was performed for the statistical analysis.

## 3. Results

### 3.1. ChP Organoid Differentiation in VWBR and Characterization

**Figure 1:**
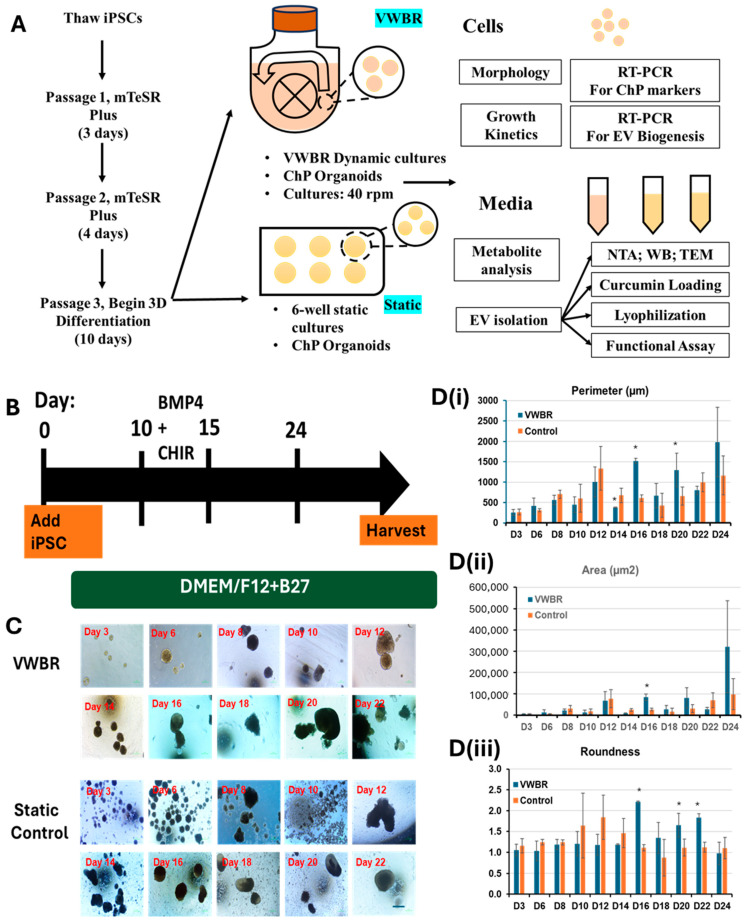
ChP organoid differentiation from hiPSCs. (**A**) Schematic illustration of study design; (**B**) ChP differentiation timeline; The arrow indicates the differentiation along the time. (**C**) the ChP organoid morphology for VWBR and static conditions during the differentiation. Scale bar: 250 µm. BMP: bone morphogenetic protein; VWBR: vertical wheel bioreactor; control: static culture. (**D**) The image analysis of aggregate size during the ChP organoid differentiation. (**i**) Perimeter; (**ii**) area; (**iii**) roundness. (n = 5–10). * indicates *p* < 0.05.

The study design is shown in Figure 1A. Throughout the differentiation, the cells were imaged every 2–3 days prior to media change to track the morphology of ChP organoids (Figure 1B). The seeded hiPSCs formed spheroids which grew larger during differentiation up to day 24 (Figure 1C). The VWBR condition showed larger organoids than the static culture at the end of differentiation. Through image analysis, the VWBR spheroids increased in size (perimeter) from ~266 µm on day 3 to ~1984 µm (i.e., 1.7-fold larger than the static group) on day 24, while the control group ranged from ~256 µm on day 3 to ~1166 µm on day 24. Until day 14, the size was comparable for the two groups. On day 16, the VWBR group showed a dramatic increase with the perimeter of ~1523 µm (i.e., 2.0–2.5 fold larger) while the control group had the values of ~605 µm. The spheroid size of the VWBR group was similar or larger than the control until the end of differentiation (Figure 1D(i)). The spheroid area showed a similar trend to the perimeter (Figure 1D(ii)). For the circularity (roundness), both groups had values around 1 (e.g., spherical) before day 10 (Figure 1D(iii)). On day 16, 20, and 22, the VWBR condition had higher values of 1.8–2.2 compared to the static control (around 1), better mimicking ChP tissue morphology.

**Figure 2:**
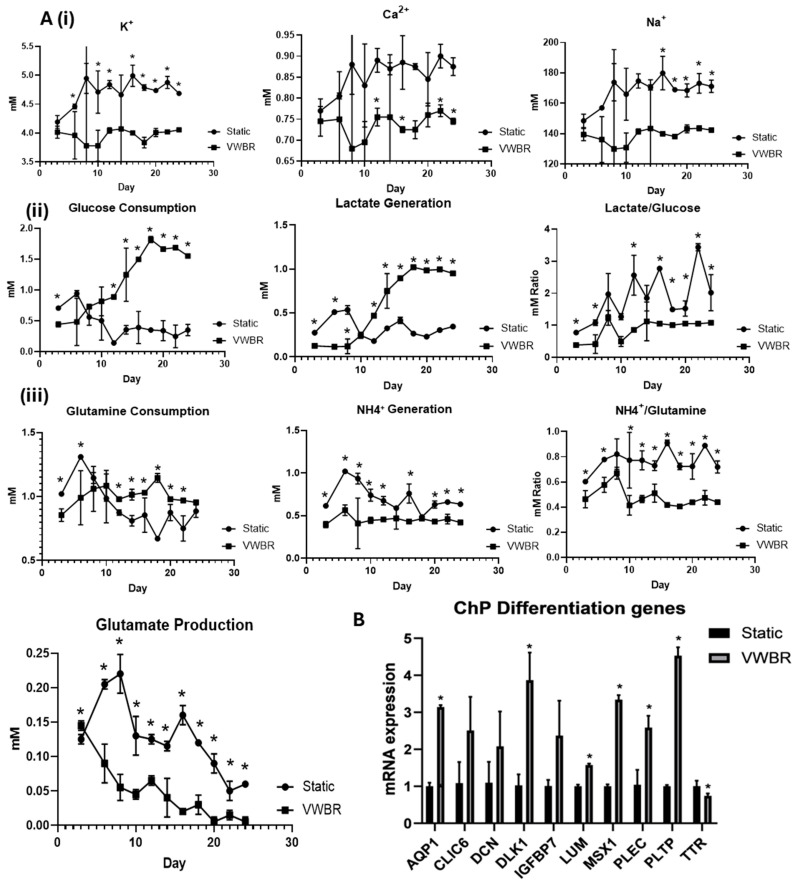
Metabolite analysis and the mRNA expression of ChP markers for VWBR and static conditions. (**A**) Metabolite analysis; (**i**) ion concentration; (**ii**) glucose consumption and lactate production, lactate to glucose mol/mol ratio; (**iii**) glutamine consumption and ammonia production, ammonia to glutamine mol/mol ratio, and glutamate generation; (**B**) mRNA expression of ChP markers was determined by RT-qPCR. VWBR: Vertical-Wheel bioreactor; N = 3. * indicates *p* < 0.05.

Metabolite analysis was performed on the spent media of the VWBR culture and the static control (Figure 2A). The K^+^, Ca^2+^, and Na^+^ concentrations were significantly lower (~4.0 mM, ~0.75 mM, and ~140 mM, respectively) in the VWBR than the static control (~4.8 mM, ~0.87 mM, and ~170 mM, respectively) over the 24-day differentiation. Glucose consumption and lactate production was significantly higher in the VWBR (>1.5 mM vs. 0.5 mM for glucose; and ~1.0 mM vs. ~0.3 mM for lactate) than the static control, particularly after day 10. The mol/mol ratio of lactate production to glucose consumption was calculated. The VWBR group had values around 1.0 (indicating aerobic metabolism) while the static control had the values of 2–3 (indicating anaerobic metabolism). The glutamine consumption was comparable for the two conditions at the beginning with the VWBR higher after day 10, while the ammonia production was higher in the static control than the VWBR condition (~0.8 mM vs. ~0.5 mM). Similarly, the glutamate production was higher in the static control than the VWBR condition, both of which decreased over the differentiation. The mol/mol ratio of ammonia production to glutamine consumption for the VWBR condition was around 0.4–0.5, while the static control had values of 0.8–0.9. All these results demonstrate the distinctly different metabolic pathways in the VWBR compared to the static control.

On day 24, the relative mRNA expression levels of various ChP markers that are typically upregulated during ChP differentiation were measured by RT-qPCR (Figure 2B). A few of these are components of the CSF, including TTR (transthyretin) and PLTP (phospholipid transfer protein), which have been detected in choroid plexus endothelial cells and possibly play a functional role in the CSF [9]. Other markers regulate signaling in the ChP, including CLIC6 (chloride intracellular channel 6), which contributes to Wnt signaling, and DLK1 (delta-like noncanonical Notch ligand 1), a transmembrane protein that regulates cell–cell contact-based signaling [9]. Additional markers are components of the cytoskeleton and extracellular matrix, including PLEC (plectin), an intermediate-filament-binding protein, DCN (Decorin), a proteoglycan closely related to biglycan, LUM (Lumican), a small leucine-rich proteoglycan (SLRP) family member [8], and IGFBP7 (insulin-like growth-factor-binding protein 7), an ECM molecule that regulates the availability of IGF and the binding of IGF to the receptors. AQP1 (aquaporin 1) is a water channel protein that is important in the secretion of CSF, and MSX1 (Msh homeobox 1) is an early regulatory factor involved in ChP development. It was observed that the VWBR group showed significantly higher expression for six of the 10 markers, including *AQP1* (~3-fold), *DLK1* (~3.8 fold), *LUM* (~1.5 fold), *MSX1* (~3.2 fold), *PLEC* (~2.5 fold), and *PLTP* (~4.5 fold), compared to the static control (Figure 2B). There is no significant difference for *CLIC6*, *DCN*, and *IGFBP7*. *TTR* expression was slightly lower (~0.6 fold) for the VWBR group compared to the static control. Taken together, these results show that the VWBR promoted ChP organoid differentiation, possibly due to the better growth factor and nutrient diffusion in the dynamic bioreactor culture microenvironment.

### 3.2. EV Biogenesis and Secretion of ChP Organoid EVs

**Figure 3:**
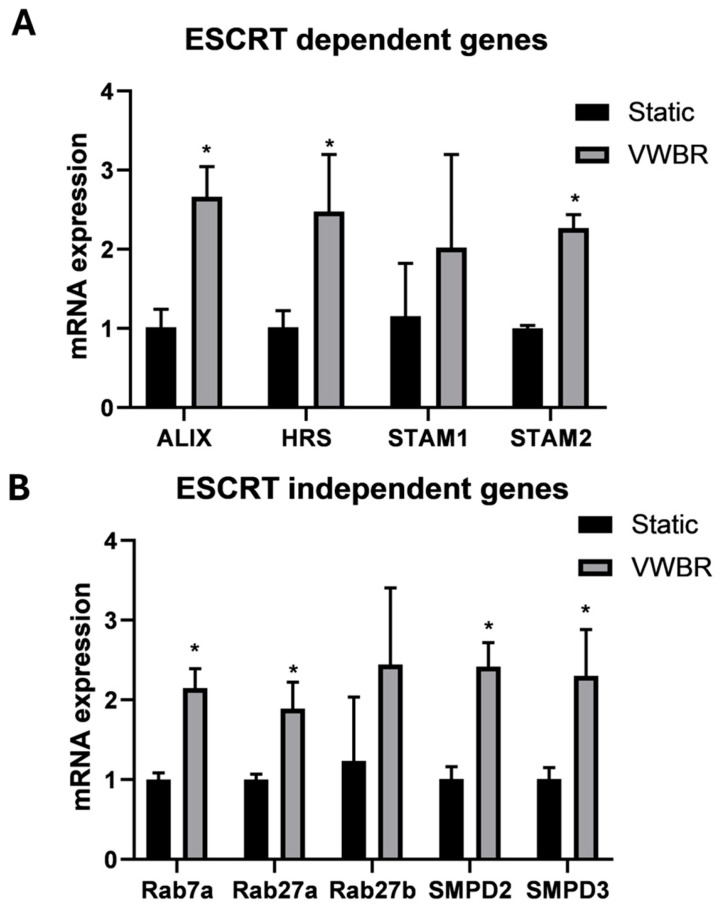
Expression of EV biogenesis genes of ChP organoids from VWBR and static conditions. mRNA expression determined by RT-qPCR for (**A**) ESCRT-dependent EV biogenesis markers; (**B**) ESCRT-independent EV biogenesis markers; VWBR: Vertical-Wheel bioreactor; ESCRT: Endosomal Sorting Complexes Required for Transport; N = 3. * indicates *p* < 0.05.

It has been recently recognized that organoids can be a reliable source for tissue-specific EVs and hold therapeutic potential [46]. Additionally, ChP EVs have been shown to be involved in neurodegenerative disease pathophysiology [47]. In this study, RT-qPCR was performed on day-24 ChP organoids for the expression of EV biogenesis markers, including both ESCRT-dependent (*ALIX*, *HRS*, *STAM1*, and *STAM2*) and -independent biogenesis genes (*Rab7a*, *Rab27a*, *Rab27b*, *SMPD2*, and *SMPD3*) (Figure 3) [48,49]. It was observed that the VWBR group showed significantly higher expression for seven of nine markers (except *STAM1* and *Rab27b* due to large standard deviations) compared to the static control. Specifically, *ALIX* showed a ~2.7-fold increase, *HRS* showed a ~2.5-fold increase, and *STAM2* showed a ~2.2-fold increase. *Rab7a*, *Rab27a*, *SMPD2*, and *SMPD3* showed about a 2–2.5-fold increase for the VWBR group compared to the static control. Taken together, the VWBR increased EV biogenesis gene expression in the ChP organoids compared to the static control, possibly as a result of the more aerobic metabolism that was observed in the bioreactor.

**Figure 4:**
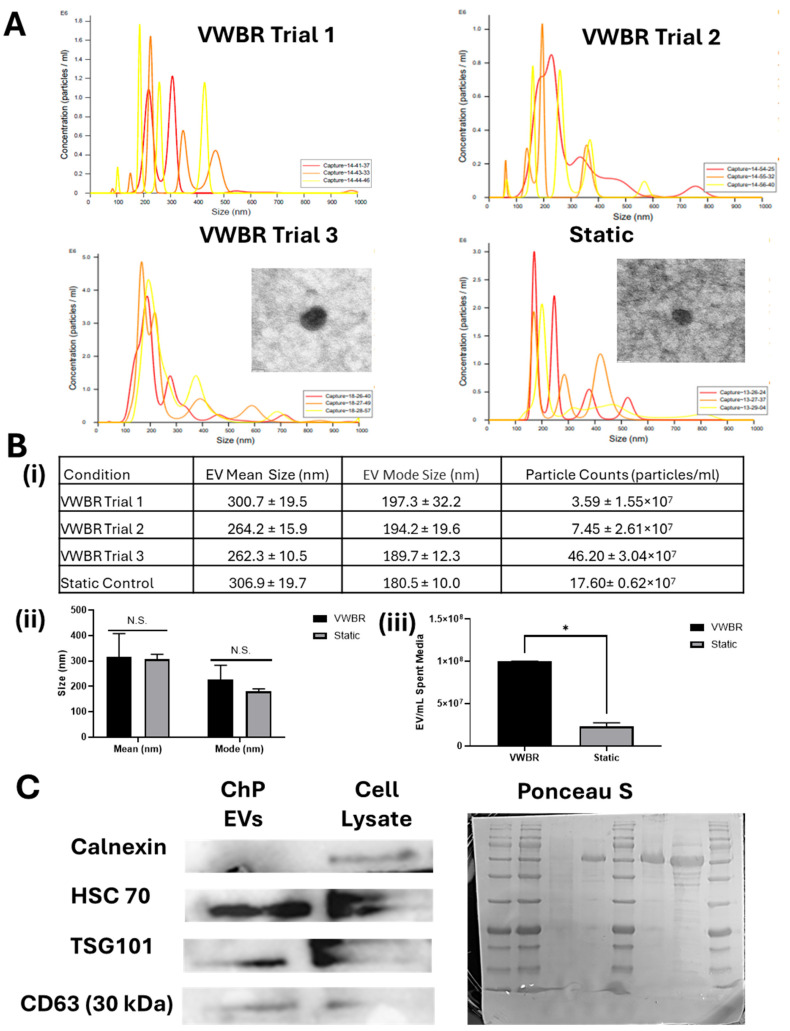
Characterizations of the isolated ChP organoid EVs. Nanoparticle tracking analysis (NTA) was performed. (**A**) Representative EV size distribution by NanoSight. The insets are the images of transmission electron microscopy. Scale bar: 60 nm. (**B**) (**i**) Tabulation of NTA results; (**ii**) EV mean size and mode size; (**iii**) EV number per mL spent media; (**C**) Western blot bands for exosomal markers. ChP EVs were tested along with the cell lysate control. Ponceau S staining showed the total protein expression. VWBR: Vertical-Wheel bioreactor; ChP: choroid plexus; EV: extracellular vesicle; N = 3. * indicates *p* < 0.05. N.S.: not significant.

EVs were isolated using the ExtraPEG-based differential ultracentrifugation method (Figure 4). The VWBR condition produced enough media for three trials of EV isolation. NTA analysis using NanoSight showed that the VWBR-derived EVs had a mode size of 197.3 ± 32.2 nm for the first trial, 194.2 ± 19.6 nm for the second trial, and 189.7 ± 12.3 nm for the third trial (Figure 4A,B). The control condition yielded EVs with a mode size of 180.5 ± 10.0 nm. The mode size is typically more accurate than mean size since it minimizes the contribution of vesicle aggregation. There was no statistical difference in the EV size for the VWBR and static conditions. TEM images showed that these EVs had a cup-shaped morphology for both conditions. The EV concentration for the first trial was 3.59 ± 1.55 × 10^7^ EVs/mL, 7.45 ± 2.61 × 10^7^ EVs/mL for the second trial, and 46.20 ± 3.04 × 10^7^ EVs/mL for the third trial based on NTA measurements. The control condition yielded a concentration of 17.6 ± 0.62 × 10^7^ EVs/mL. The directly measured EV concentration was normalized to EV number per mL spent medium (Figure 4B). The VWBR condition produced four-fold-higher EVs per mL medium than the static control, consistent with the upregulated gene expression of EV biogenesis. The Western blot assay showed that the EVs of ChP organoids from the VWBR as well as the cell sample expressed positive exosomal markers HSC70, TSG101, and CD63 (Figure 4C and Supplementary Figure S1). The negative exosomal marker Calnexin was present in the cells but not expressed in the EVs, as expected. The static control did not have sufficient EVs for the Western blot assay. Taken together, these results show that the VWBR promoted EV biogenesis and secretion of ChP organoids compared to the static control.

### 3.3. EV Loading with Curcumin and the EV Lyophilization

EV loading was performed via various methods, using adipose tissue-derived hMSC EVs for process optimization (Supplementary Figure S2). EV size and concentration were analyzed via NTA before and after each loading method (Supplementary Figure S2). EVs prior to loading display a size of 100.3 ± 22.9 nm (sonication) and 189.5 ± 78.1 nm (incubation and freeze–thaw) (Supplementary Figure S3). After loading, EV samples that underwent sonication had a size of 120.8 ± 20.9 nm and EVs that underwent incubation and freeze–thaw had sizes of 111.6 ± 47.7 nm and 122.5 ± 71.1 nm, respectively. For EV yield, sonication showed 7.0 ± 0.8 × 10^8^ EVs/mL in 2 mL before loading and 1.7 × 10^9^ EVs/mL in 0.5 mL after loading (yield: 61%). The incubation method showed 4.3 ± 0.4 × 10^7^ EVs/mL in 2 mL before loading and 4.1 ± 0.4 × 10^7^ EVs/mL in 1.5 mL after loading (yield: 72%). The freeze–thaw method showed the process yield of 74%. The tabulated results were summarized in Supplementary Figure S3. To measure the loading efficiency, the curcumin solution was diluted from 100% to 0% to generate a standard curve (Supplementary Figure S4). The values of the samples were then measured by comparing to the standard curve to determine the loading efficiency. For adipose tissue-derived hMSC EVs loaded via sonication, for example, the sample value was 145, corresponding to a concentration of 0.0059 mM. The loading efficiency of this trial was 34.8%. The loading efficiency of ChP organoid-derived EVs loaded via sonication was determined to be 30.5%. For adipose tissue-derived hMSC EVs loaded via incubation, a loading efficiency of 33.4% was observed. For EVs loaded via freeze–thaw, the loading efficiency was 18.9%.

**Figure 5:**
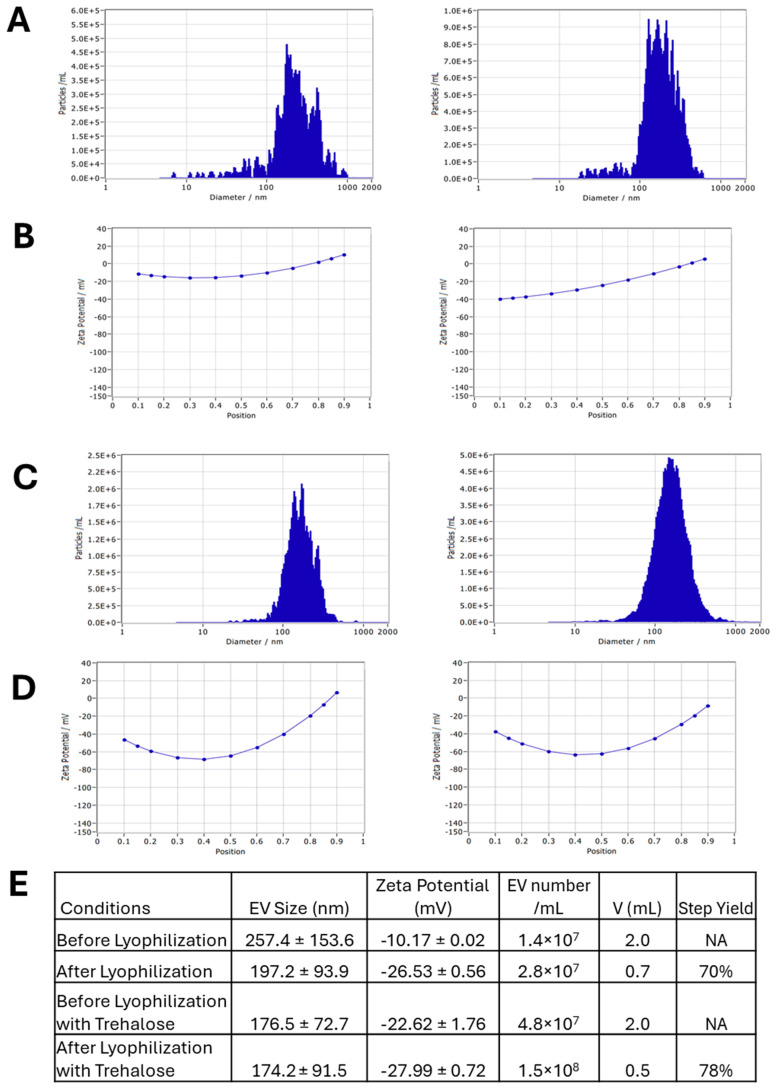
Impact of lyophilization on size and zeta potential of hMSC EVs. (**A**) Size before (left) and after (right) lyophilization; (**B**) zeta potential before (left) and after (right) lyophilization; (**C**) size before (left) and after (right) lyophilization with trehalose; (**D**) zeta potential before (left) and after (right) lyophilization with trehalose. (**E**) The summary table for EV lyophilization effects. EV: extracellular vesicle. NA: Not applicable.

The EV lyophilization process is shown in Supplementary Figure S5A. The protein content of the adipose tissue-derived hMSC-derived EVs was quantified using a Bradford assay to determine the amount of trehalose to be used during lyophilization (Supplementary Figure S5B). The ratio of trehalose to EV protein content should be 1:1 by weight for proper lyoprotection. Based on the standard curve, it was determined that the protein concentration of an EV sample was 155.0 μg/mL and another EV sample had a value of 171.1 μg/mL. EV size and concentration were analyzed via NTA before and after lyophilization (Figure 5). Before lyophilization, EVs displayed a slightly large size of 257.4 ± 153.6 nm and high zeta potential of −10.17 ± 0.02 mV (Figure 5A,B). This is likely due to EV clumping during NTA. After lyophilization, EVs displayed a typical size of 197.2 ± 93.9 nm and zeta potential of −26.53 ± 0.56 mV (Figure 5A,B). Lyophilization was also performed in the presence of trehalose (Figure 5C,D) [50,51,52,53,54]. A similar trend was observed between fresh EVs and lyophilized EVs, with a respective size of 176.5 ± 72.7 nm and 174.2 ± 91.5 nm and respective zeta potential of −22.62 ± 1.76 mV and −27.99 ± 0.72 mV (Figure 5C,D). For the EV yield, the lyophilization–re-hydration process resulted in a 70% step yield based on the measured EV concentration and the sample volume. In the presence of trehalose, the step yield of lyophilization and re-hydration was 78% (Figure 5E). Overall, these results suggest that lyophilization does not negatively affect EV size and stability.

### 3.4. Functional Testing for Lyophilized Curcumin-Loaded ChP Organoid EVs

**Figure 6:**
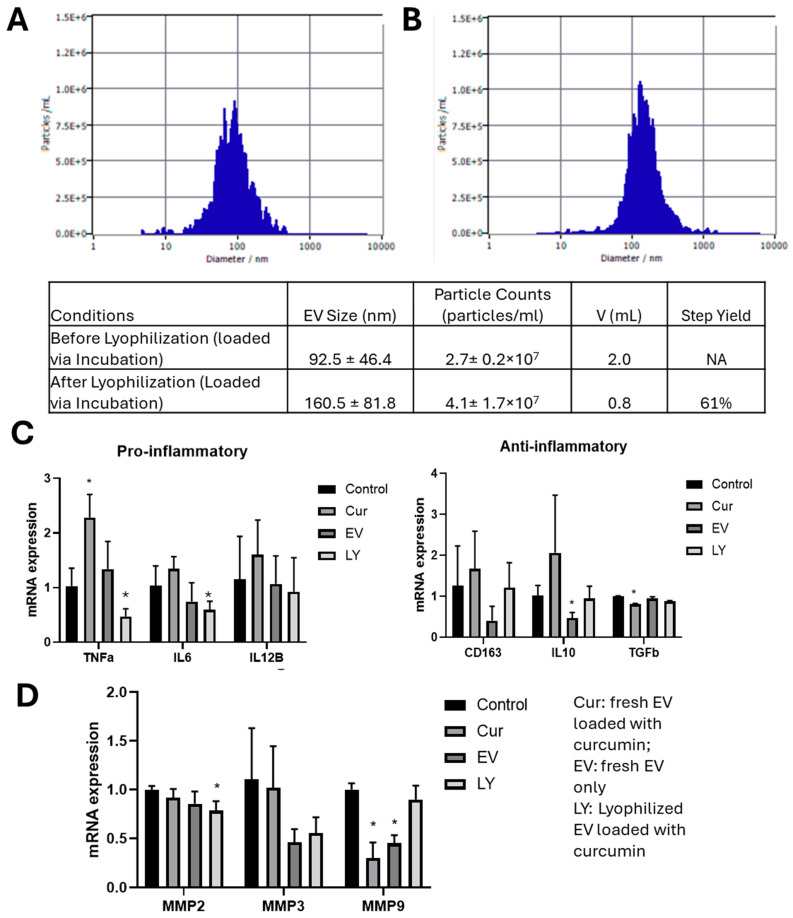
In vitro functional testing for lyophilized curcumin-loaded ChP organoid EVs. Size and concentration before (**A**) and after (**B**) lyophilization, determined by zeta view. Aβ42 oligomers were added to ChP organoids for two days. After this, curcumin-loaded EVs, unloaded EVs, and lyophilized curcumin-loaded EVs were introduced to the cultures for two days. The cells were harvested for RT-qPCR analysis. (**C**) *TNF-α*, *IL-6*, and *IL-12β* for proinflammatory response and *TGFβ*, *IL-10*, and *CD163* for anti-inflammatory response. (**D**) MMP2, MMP3, MMP9 expression (n = 3). * indicates *p* < 0.05. NA: Not applicable.

To evaluate the effect of loaded EVs on recipient cell viability, a Live/Dead assay was performed on the hiPSC-derived neural progenitor cells treated with adipose tissue-derived hMSC EVs and the loaded EVs. High viability was observed at 86–91% determined by flow cytometry for all the groups (Supplementary Figure S6A). Therefore, curcumin-loaded EVs did not affect cell viability. To evaluate how EV bioactivity is affected by loading and lyophilization, an inflammatory model employing Aβ42 oligomers was used to inflame hiPSC-derived neural progenitor cells. Aβ42 oligomers (1 μM) were added to the cultures for 48 h before treating with EVs for 48 h and the cell viability was analyzed via a Live/Dead assay (Supplementary Figure S6B). Based on the images, the viability was comparable for the lyophilized EVs, the lyophilized EVs with trehalose, and the fresh EVs, which were higher than the control that had no EV treatment. Based on these results, ChP organoid EVs were evaluated for the loading and lyophilization process. ChP organoid-derived EVs were loaded with curcumin via incubation and subsequentially lyophilized. Before lyophilization, EVs displayed a typical size of 92.5 ± 46.4 nm. After lyophilization, an increase in EV size to 160.5 ± 81.8 nm was observed, but the difference was not statically significant (Figure 6A,B). This observation may be due to the EV aggregation during NTA measurement. Based on the measured EV concentrations, the step yield of loading and lyophilization was 61%.

For functional testing, Aβ42 oligomers (1 μM) were added to the replated ChP organoids for 48 h before treating with EVs. The groups included fresh EVs, fresh EVs loaded with curcumin, and lyophilized EVs loaded with curcumin. The cells were harvested for RT-qPCR analysis of pro-inflammatory (*TNF-a*, *IL-6*, *IL12β*) and anti-inflammatory (*CD163*, *IL10*, and *TGFβ*) markers, as well as extracellular matrix (ECM) remodeling proteins (*MMP2*, *MMP3*, and *MMP9*) (Figure 6C). The curcumin-loaded EVs showed significant upregulation of *TNF-α* while the lyophilized curcumin-loaded EVs showed downregulation of *TNF-α* and *IL-6*, indicating the effect to reduce pro-inflammation. For the anti-inflammatory markers, the fresh EVs showed a low expression of *IL-10*, but other conditions did not significantly alter the marker expression. Aβ42 oligomers are known to activate matrix metalloproteinases (MMPs); therefore, the expression of *MMP2*, *MMP3*, and *MMP9* was also determined. The condition of lyophilized curcumin-loaded EVs showed similar *MMP2* expression compared to fresh EVs and curcumin-loaded EVs, while showing slightly lower expression compared to the control. Similar *MMP3* and *MMP9* expression was observed for the condition of lyophilized curcumin-loaded EVs compared to the control. The conditions of curcumin-loaded EVs and the fresh EVs showed a low expression of *MMP9*. Taken together, lyophilized curcumin-loaded EVs preserve the functional properties of the ChP organoid EVs, alleviating neuro-inflammation induced by exposure to Aβ42 oligomers.

## 4. Discussion

### 4.1. ChP Organoid Differentiation in VWBR

In this study, human ChP organoids were derived from hiPSCs in the dynamic microenvironment of the VWBR. The culture was compared to a static aggregate control culture. The two culture conditions had the same initial seeding density of 1 × 10^5^ cells/mL, but over time the VWBR conditions had a larger organoid size than the static control. Consistently, larger spheroids in the bioreactor cultures usually indicate better nutrient transfer and growth. These observations were also consistent with the significantly increased glucose consumption and lactate production for the VWBR condition compared to the static condition based on the results in this study. Additionally, in this study, the lactate-to-glucose ratio for the VWBR was consistently low (~1.0) over the differentiation period, which indicated aerobic metabolism and the decreased chances of cellular necrosis within the organoid core, while the static condition had values of 2–3 showing anerobic metabolism due to diffusion limitation. The ratio of lactate to glucose in metabolites is influenced by factors such as the rate of anaerobic glycolysis, oxygen availability, etc. The bioreactor condition promotes more aerobic metabolism due to better oxygen transfer than the static condition, which correlates with shifting to oxidative phosphorylation, or aerobic glycolysis, and the enhanced differentiation. These results are consistent with other studies on VWBRs that focus on hiPSC expansion rather than differentiation, which find that cell expansion increased 10-fold within the VWBR when compared to static conditions [55,56]. Another important implication of the improved culture outcomes for the VWBR is that this system is also scalable (up to 80–500 L), with the potential to provide a more cost-effective way to produce large amounts of ChP organoids as well as their therapeutically relevant products (EVs and other execrated proteins and fluids).

Furthermore, the bioreactor culture condition in this study was shown to be beneficial for ChP organoid differentiation. Analysis of differentiation markers in these bioreactor-differentiated organoids by RT-qPCR at day 24 revealed a significant upregulation of most ChP differentiation genes tested except *TTR*. This upregulation in differentiation genes is consistent with our observations for other types of organoids grown in the VWBR [29,34]. Our previous studies demonstrated that the culture and differentiation of human forebrain spheroids in a VWBR resulted in the upregulation of human forebrain differentiation markers compared to the static culture [29,34]. In addition, the blood vessel organoid differentiation in a VWBR was also promoted compared to the static culture, as shown in our previous study [57]. Here, this study shows that the VWBRs can be used to promote the differentiation of choroid plexus organoids from hiPSCs. The differentiated choroid plexus organoids, however, yield some limitations. Due to the homogeneity of the organoids produced, they may not be fully representative of the more complex cell populations within the choroid plexus, which contains a plethora of other cell types such as ependymal cells, pericytes, and progenitor-like cells. Co-cultures or assembloids would be needed to generate a more representative model. Differentiation in other 3D biomaterials may also be used to generate good 3D models [58].

### 4.2. EV Biogenesis of ChP Organoids in VWBR

The EVs that ChP organoids produce as well as the other secreted products are of great interest because of their therapeutic relevance [59,60,61]. The production of both the organoids and their secreted products using bioreactors could provide a scalable and cost-effective approach for potential preclinical and clinical applications. However, it is important to characterize these secreted products, particularly under the dynamic culture conditions. In this study, ChP organoid EVs from the VWBR and static conditions had a mode size of ~190 nm, indicating that the dynamic environment did not significantly change the EV size. The VWBR conditions showed the four-fold-higher EV numbers per mL spent medium when compared to the static condition. The results are consistent with the expression of ESCRT-dependent and -independent EV biogenesis genes tested by RT-qPCR as well as our previous studies [34,57]. This study provides further evidence that VWBRs greatly promote EV production from ChP organoids, similar to other types of stem cells [28,29,30,31] as well as other types of spheroids and organoids [29,34,57].

The VWBR culture provides further evidence for the impact of shear forces on EV production. Shear has been proposed to help cleave EVs once ligand binding creates membrane tethers [62,63]. The VWBR has efficient mixing at low shear stress, about 10-fold lower compared to traditional stirred tank bioreactors (0.1–0.3 dyn/cm^2^ vs. 1–3 dyn/cm^2^, respectively). The low shear stress levels in VWBRs should have minimal influence on the production of large EVs, which can be shed from the cells under high shear stress. It has also been proposed that high intracellular levels of Ca^2+^ are related to EV production in adipose tissue-derived hMSCs [64,65]. Here, this study observed lower Ca^2+^ concentrations in the culture media of dynamic conditions, consistent with our previous work in forebrain spheroid differentiation and indicative of higher intracellular ion levels, thus promoting EV generation [29,34]. Furthermore, the improvements in aerobic metabolism observed in the VWBR culture may also contribute to increases in EV production. EV biogenesis is significantly influenced by cellular metabolism, for example, increased glycolysis can promote EV production, and it is possible that the bioreactor culture uses aerobic glycolysis. On the other hand, the shear stress in the bioreactor can promote EV biogenesis. The observed EV secretion in the bioreactors may be the consequence of multiple mechanisms. Overall, the VWBR system provides a promising approach for the scalable differentiation of ChP organoids, as well as the production of ChP organoid-derived EVs.

In addition, this study used an hiPSC line from a de-identified donor. The possible influence of the iPSC line source (e.g., donor sex) may need to be evaluated using male- or female-derived iPSCs in the future.

### 4.3. Loaded ChP Organoid EVs as Neurological Therapeutics

Research into EV-based applications for neurological therapeutics has only recently been investigated. The natural composition of nanosized EVs, engagement in intercellular interactions, and unique ability to cross the blood brain barrier make EVs attractive candidates for drug delivery to the brain. Few studies have measured the loading efficiency of ChP organoid EVs with curcumin and demonstrated the drug’s ability to reduce inflammation post-delivery. Curcumin has several medicinal properties, including antioxidant, anti-carcinogenic, and anti-inflammatory abilities [19,20,21,22], while most previous studies focused on loading hMSC-derived EVs. This study investigated the loading of ChP organoid-derived EVs, evaluated three different loading methods, and tested the anti-inflammatory properties of curcumin-loaded EVs.

Incubation is the most straightforward and simplest way for loading EVs. For this method, the cargo is incubated with EVs or EV-secreting cells. Driven by the concentration gradient, the cargo diffuses into the EVs [66]. For sonication, EVs are mixed with the cargo and sonicated with a probe sonicator. This process causes the EV membrane to rupture and reform, thus encapsulating the cargo. This method has previously been used to load EVs with siRNAs for treatment on an animal model of breast cancer [67]. Sonication is easily scalable and could be used to load EVs with relatively bulky molecules. Freeze–thaw cycling is performed by alternating cycles of temperature shock, with incubation of EVs with the cargo at room temperature and then frozen at −80 °C. Repeating the cycles has shown to increase loading efficiency. This method utilizes the expansion of water upon freezing to cause cell membrane permeabilization. Typically, this method is used to encapsulate drug molecules in lipid bilayer vesicles [67,68]. Electroporation is another possible EV loading method that utilizes an electrical field. When applied to EVs, pores are created in the lipid bilayer membrane to facilitate EV loading. Similar to sonication, this method is useful for loading large macromolecules such as siRNAs to knockdown therapeutic targets in Alzheimer’s disease [69].

In this study, curcumin was firstly loaded into adipose tissue-derived hMSC-derived EVs to optimize the process and develop a loading protocol that was then applied to ChP organoid EVs [70]. Curcumin has been reported to prevent the cellular senescence of adipose tissue-derived hMSCs [71]. When loading the hMSC EVs via sonication, the loading efficiencies were found to range between 12.7–34.8%. This is in line with current literature that has shown sonication to have a loading efficiency of 8–30% [72]. The adipose tissue-derived hMSC EVs were also loaded with curcumin via incubation with 33% loading efficiency and freeze–thaw, which had a loading efficiency of ~19%. Studies have shown low-power sonication to cause damage to the EV membrane, thus causing a decrease in drug uptake and ultimately a decrease in loading efficiency [73]. In this study, ChP organoid EVs were loaded via the same mechanisms used to load hMSC EVs. Using sonication, ~30% loading efficiency for ChP EVs was observed. Sonication may cause damage to the membrane of ChP EVs similar to adipose tissue-derived hMSC EVs. The step loss can be minimized to improve the sonication process. Taken together, all the three methods of incubation, sonication, and freeze–thaw can be used to load EVs with curcumin as a proof-of-concept process.

### 4.4. EV Lyophilization for Preservation

Lyophilizing EVs presents a number of benefits over the frozen storage in potential preclinical and clinical applications. Lyophilization allows EVs to be stored at room temperature without the degradation that would occur with storage in an aqueous buffer, reducing the cost of the storage and transportation of EVs and eliminating the negative effects of ice crystal formation during freeze–thaw cycling [51,52,74,75]. Furthermore, lyophilized EV powder can be quickly re-hydrated in water or a buffer whenever needed. Lyophilized storage of EVs can maintain the particle size, improve zeta potential (propensity of particles to aggregate), improve cellular uptake efficiency, and improve biological activity of EVs at a level comparable to fresh EVs. Despite these many benefits, lyophilization procedures conducted without lyoprotectants, with the direct freeze-drying procedure, can induce large mechanical stresses or cause phase transitions in the lipid membranes of EVs [76,77]. Therefore, lyoprotectants are usually included in the formulation of EV solution.

The lyophilization process may exert freezing stress on the EVs, leading to mechanical damage due to ice crystal formation, exposure to ice–liquid interfaces, increased iron strength, and pH shifts induced by salt precipitation [77]. The re-hydration process may cause damage due to the swelling of the amphiphilic molecules in the vesicle bilayer. These influences could be cell type-specific. The presence of trehalose is to protect EVs from these damages, and it has been demonstrated that trehalose had better protective effects on the EVs than mannitol and PEG400 [78]. When lyophilized, the EVs in the solution containing trehalose become suspended in a glassy matrix that is able to stabilize their surface proteins and maintain native configuration, separate and protect individual EVs from mechanical stresses, and reduce the total water content of the lyophilized product [52]. Trehalose in particular is favored due to the high glass transition temperature (Tg) of its matrix in the lyophilized state. Other EV cryoprotectants include sucrose and surface-active stabilizers [77]. The pH shifts during lyophilization may destabilize surface or membrane proteins of the EVs as well as affect the zeta potential and colloidal interactions of the EVs. In our study, the zeta potential was around −26 to −28 mV, which was within the optimal range, and higher particle dispersion allows for better drug delivery. Further improvement can be made by adding surfactants such as polysorbate 80 or antioxidants such as polyvinylpyrrolidone 40 (PVP40), which provide additional steric stabilization to the EVs and allow them to retain their bioactivity for longer periods of time when in storage [51,52]. In particular, further investigation is required to evaluate the effects of the loading and lyophilization process on the natural EV cargo profiles (i.e., proteins, miRNAs, and lipids) through multi-omics analysis.

Nonetheless, the biological activity of the lyophilized curcumin-loaded ChP organoid EVs was evaluated as a proof-of-concept study along with fresh EVs and fresh curcumin-loaded EVs in a neuroinflammation model induced by amyloid beta oligomers. The lyophilized EVs downregulated *TNFα* and *IL-6* expression, indicating the effect of reducing pro-inflammatory markers. The anti-inflammatory effect was similar to or better than the fresh EVs. The lyophilized EVs also showed similar effects on the gene expression of matrix remodeling enzymes compared to fresh EVs. To the best of our knowledge, there are no previous studies concerning the lyophilization of loaded ChP organoid EVs and their biological function. Further, thorough investigation is required to evaluate the function of the lyophilized ChP organoid EVs toward potential in vivo study.

## 5. Conclusions

This study investigated ChP organoid differentiation and the EV secretion in a Vertical-Wheel bioreactor. The VWBR results in more aerobic metabolism and active glucose and glutamine consumption, demonstrating distinctly different metabolic pathways compared to the static control. Consequently, the ChP markers and ESCRT-dependent and -independent EV biogenesis genes were significantly upregulated in the VWBR. The VWBR condition produced four-fold-higher EVs per mL medium than the static control, consistent with the upregulated gene expression of EV biogenesis. This study also assessed the loading of curcumin into the ChP organoid-derived EVs, and the loading process did not significantly affect the EV size with the process yields around 60–74%. For long-term storage, EV lyophilization was performed in the presence of trehalose. The EV size did not show a significant difference pre- vs. post-lyophilization. The curcumin-loaded ChP organoid-derived EVs were lyophilized and re-hydrated. Alleviating neuro-inflammation induced by exposure to Aβ42 oligomers was demonstrated for the treatment by re-hydrated EVs. This study demonstrates a scalable bioreactor system to promote ChP organoid differentiation and the EV secretion for potential applications in treating ischemic stroke and Alzheimer’s disease. This study also demonstrates the feasibility of drug loading and lyophilization of the ChP organoid EVs to develop cell-free therapeutics for treating neural inflammation in neurological disorders.

## Data Availability

The datasets generated during and/or analyzed during the current study are available from the corresponding authors on reasonable request.

## Supplementary Materials

The following supporting information can be downloaded at https://www.mdpi.com/article/10.3390/biomedicines13051069/s1. Figure S1: The full western blot image for Figure 4C; Figure S2: Schematic illustration of EV loading using different methods; Figure S3: Impact of loading methods on EV size and yield; Figure S4: Determination of EV loading efficiency; Figure S5: Illustration of EV lyophilization process; Figure S6: In vitro functional testing for loaded or lyophilized hMSC EVs; Table S1: Primer information for RT-qPCR analysis; Table S2: A list of antibodies; Table S3: Abbreviation list.

## Abbreviations

The abbreviations are shown in Supplementary Table S3.
