# Supplementary material for: Biomanufacturing and Curcumin-Loading of Human Choroid Plexus Organoid-Derived Extracellular Vesicles from a Vertical-Wheel Bioreactor to Alleviate Neuro-Inflammation
Source: Biomedicines. 2025 Apr 28;13(5):1069. doi: 10.3390/biomedicines13051069 (PMC12109122; doi:10.3390/biomedicines13051069)
Supplement: Supplementary file 1 [file biomedicines-13-01069-s001.zip › biomedicines-3569424-supplementary.pdf]

biomedicines

MDPI

Supplementary Materials

Biomanufacturing and Curcumin-Loading of Human Choroid Plexus Organoid-Derived Extracellular Vesicles from a Vertical-Wheel Bioreactor to Alleviate Neuro-Inflammation

Justice Ene [1,†], Laureana Muok [1,†], Vanessa Gonzalez [1], Nicolas Sanchez [1], Aakash Nathani [2], Falak Syed [1], Zixiang Leonardo Liu [1], Mandip Singh [2], Tristan Driscoll [1] and Yan Li [1,*]

[1] Department of Chemical and Biomedical Engineering, FAMU-FSU College of Engineering, Florida State University, Tallahassee, FL 32310, USA; je17d@fsu.edu (J.E.); laureana1.muok@famu.edu (L.M.); vgonzalez2@fsu.edu (V.G.); ns20d@fsu.edu (N.S.); fs23i@fsu.edu (F.S.); leo.liu@eng.famu.fsu.edu (Z.L.L.); tdriscoll2@eng.famu.fsu.edu (T.D.)

[2] College of Pharmacy and Pharmaceutical Sciences, Florida A&M University, Tallahassee, FL 32310, USA; akash.nathani96@gmail.com (A.N.); mandip.sachdeva@famu.edu (M.S.)

* Correspondence: yli4@fsu.edu

† These two authors contribute equally to this work.

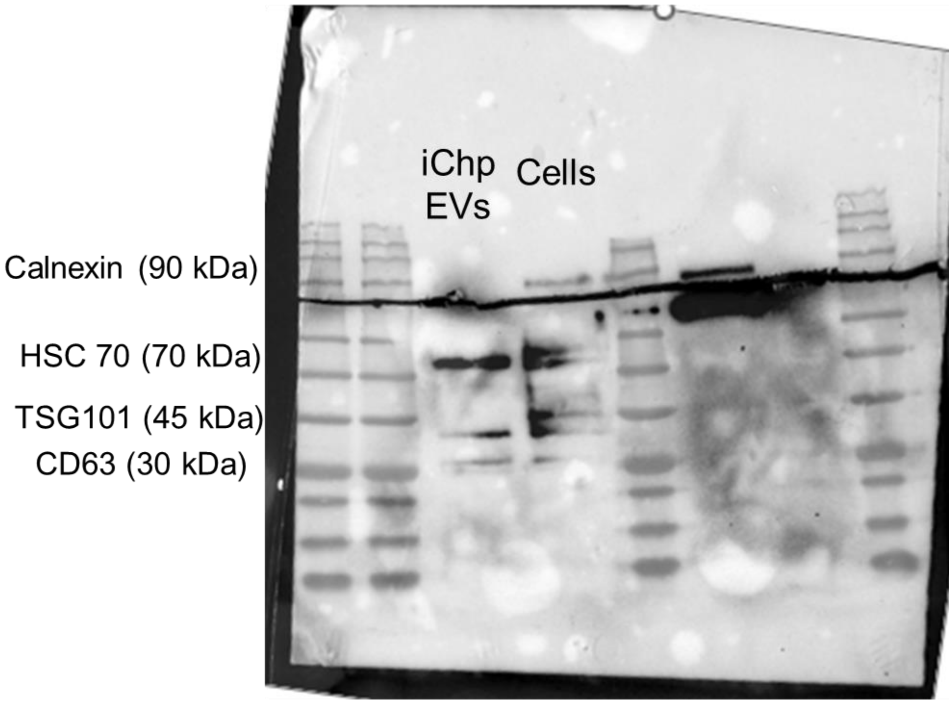

Figure S1. The full western blot image for Figure 4C. .

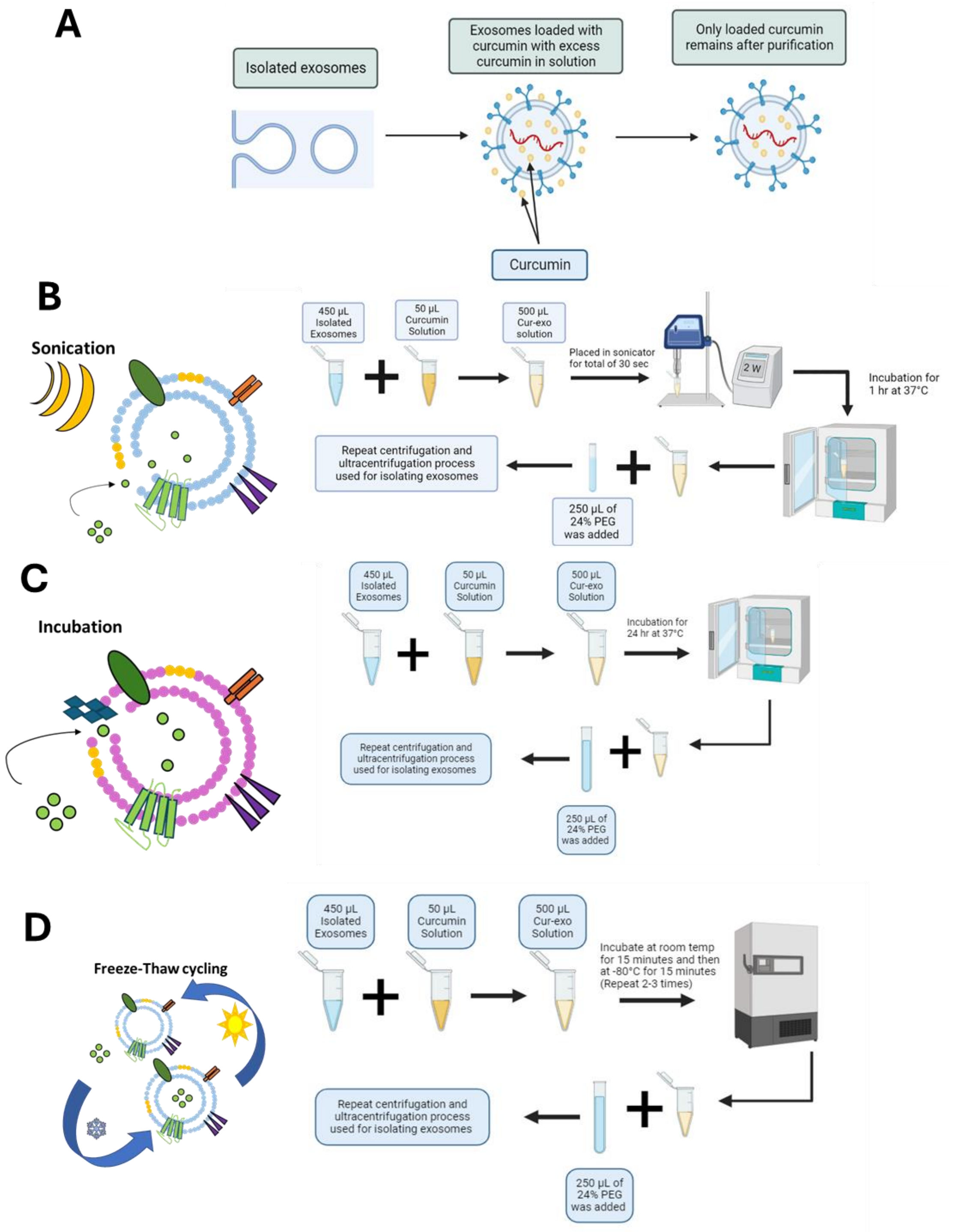

Figure S2. Schematic illustration of EV loading using different methods. (A) Illustration of EV loading with curcumin. Isolated EVs are loaded with curcumin in solution. After purification, only EVs loaded with curcumin remains. (B) Schematic illustration of EV loading using sonication; (C) Schematic illustration of EV loading using incubation; (D) Schematic illustration of EV loading using freeze-thaw.

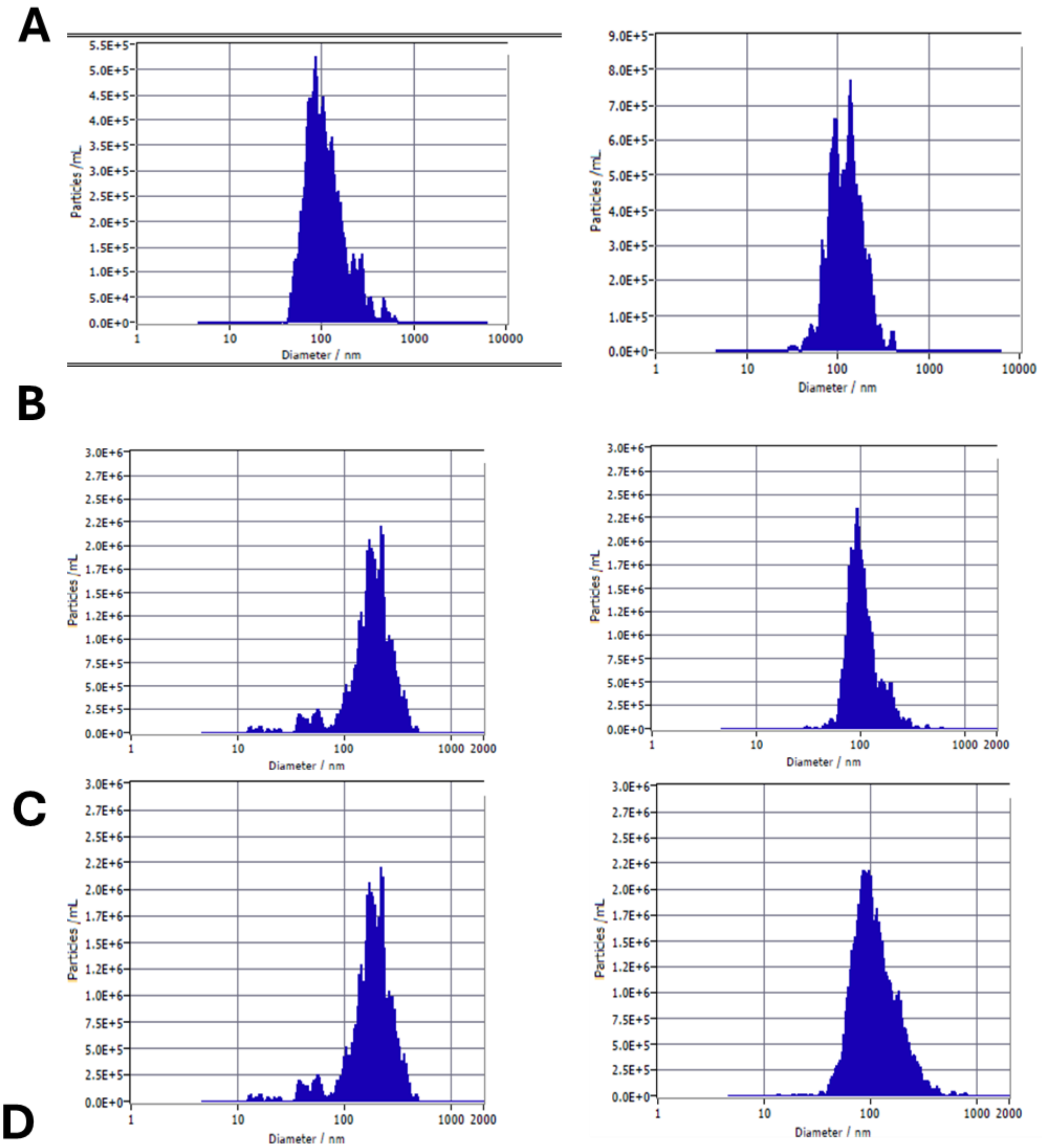

D

Conditions	EV Size (nm)	EV number/mL	V (mL)	Step Yield
Before Sonication	100.3 ± 22.9	$7.0 \pm 0.8 * 10^{8}$	2.0	NA
After Sonication	120.8 ± 20.9	$1.7 \pm 0.8 * 10^{9}$	0.5	61%
Before Incubation and Freeze-Thaw	189.5 ± 78.1	$4.3 \pm 0.4 * 10^{7}$	2.0	NA
After Incubation	111.6 ± 47.7	$4.1 \pm 0.2 * 10^{7}$	1.5	71%
After Freeze-Thaw	122.5 ± 71.1	$6.4 \pm 0.4 * 10^{7}$	1.0	74%

Figure S3. Impact of loading methods on EV size and yield. (A) Size before (left) and after (right) sonication on hMSC EVs; (B) Size before (left) and after (right) incubation on EVs; (C) Size before (left) and after (right) freeze-thaw on EVs. (D) The summary table of EV size and concentration using different loading methods from NTA measurements.

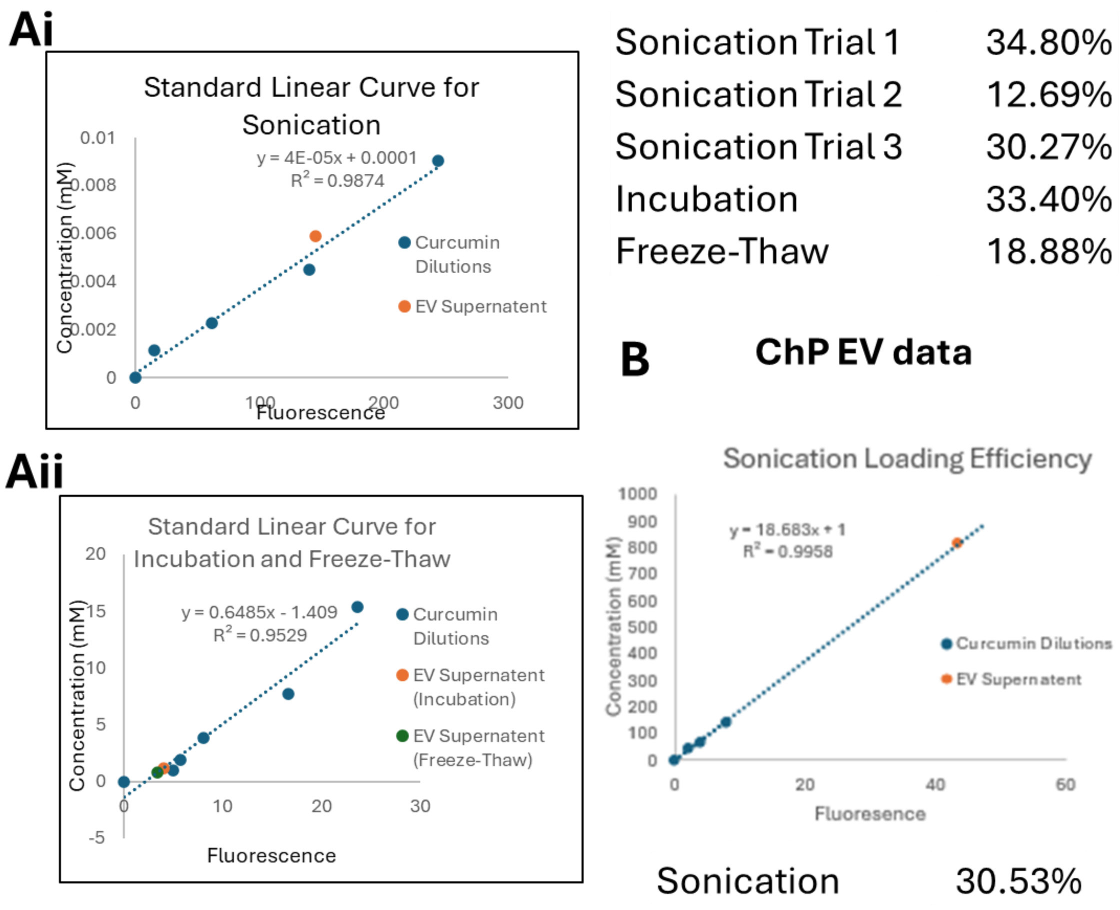

Figure S4. Determination of EV loading efficiency. Standard curves of curcumin solution were determined, and the sample values were compared to the standard curves to obtain the concentrations. (A) (i) Sonication using hMSC EVs. (ii) Incubation and Freeze-Thaw using hMSC EVs; (B) Sonication loading of ChP organoid EVs.

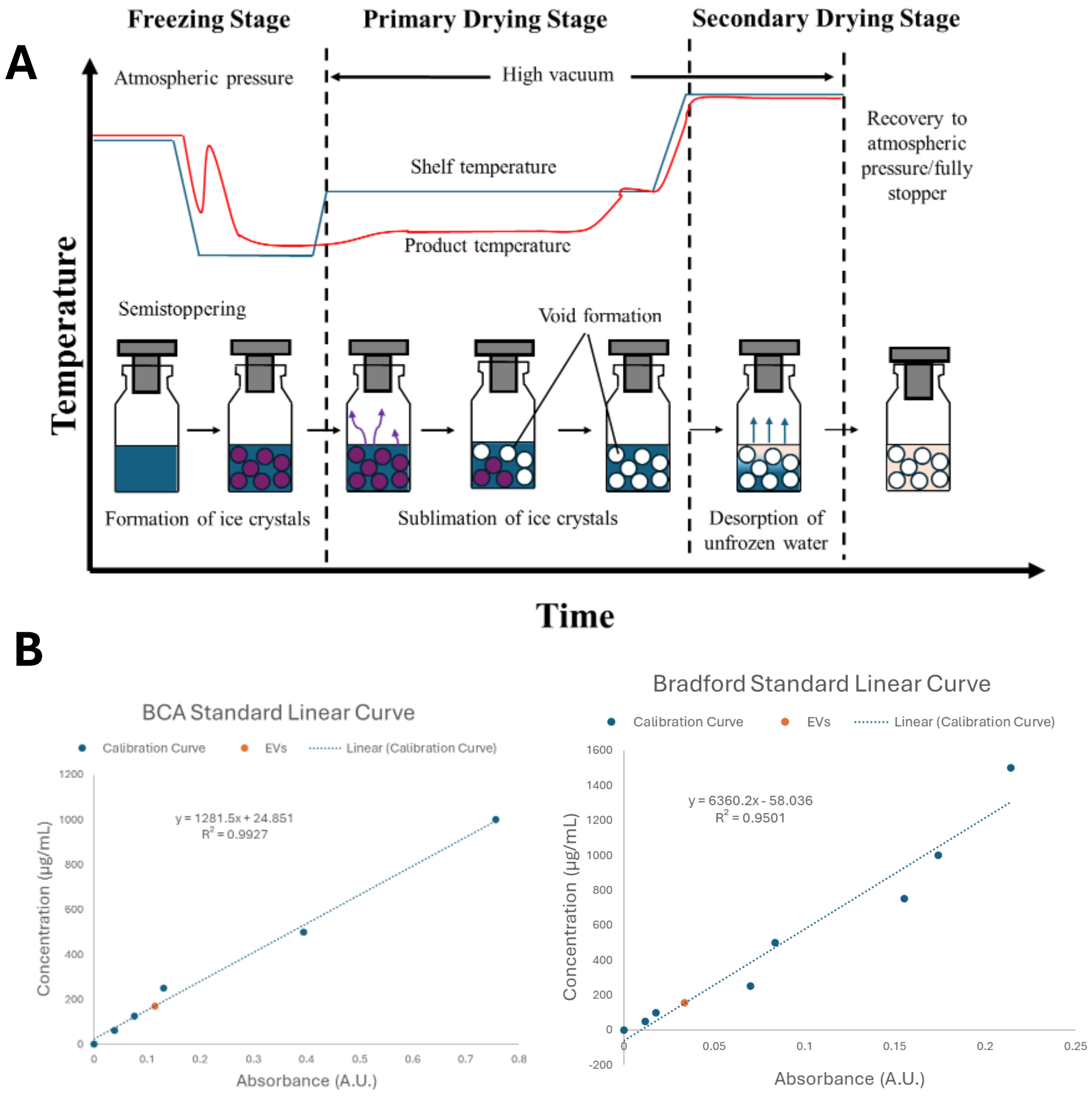

Figure S5. Illustration of EV lyophilization process. (A) Freeze-drying program; (B) Protein quantification of EVs to determine the EV lyophilization condition with the required trehalose amount.

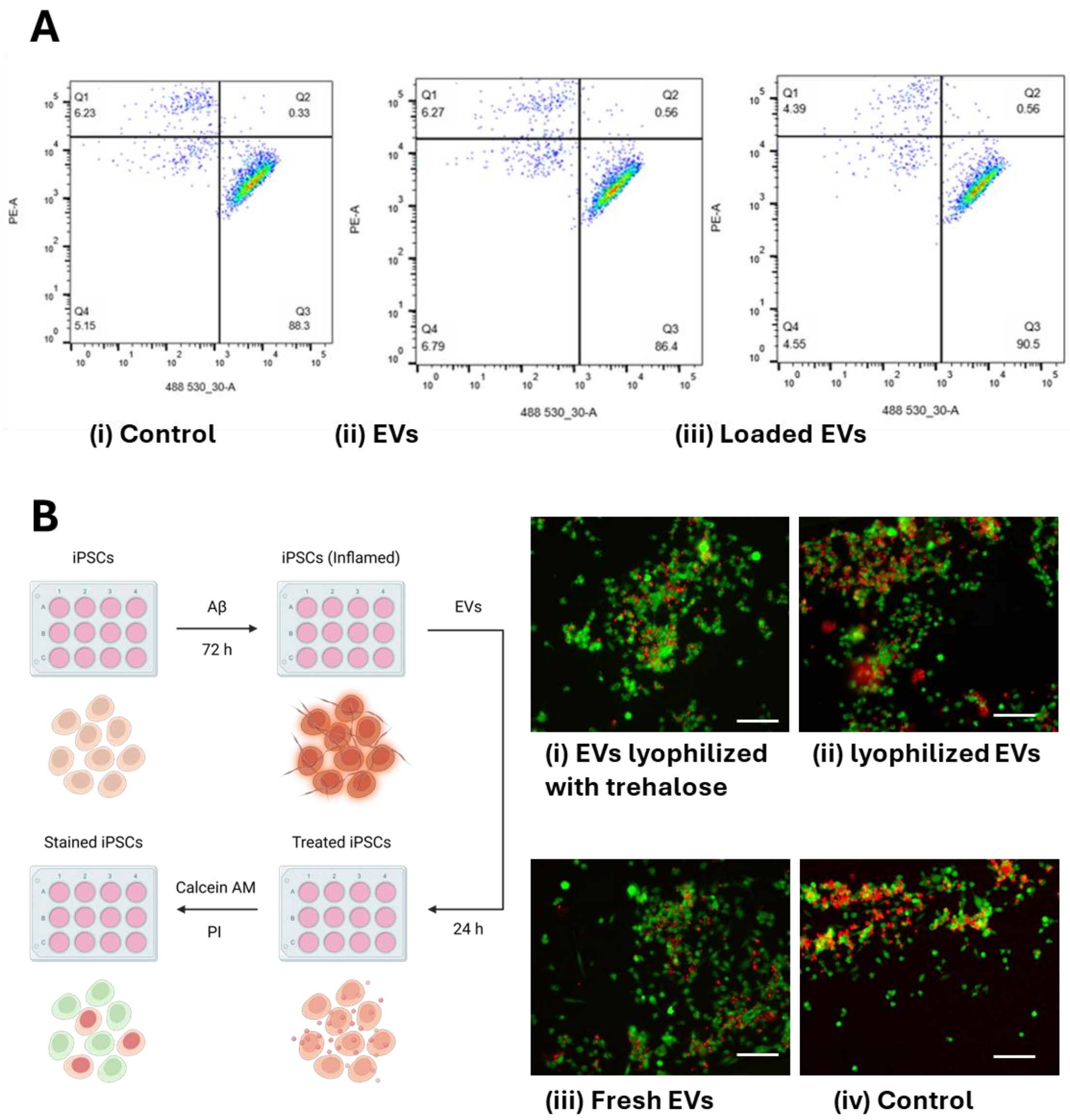

Figure S6. ***In vitro*** **functional testing for loaded or lyophilized hMSC EVs**. (A) Live/dead flow cytometry assay for the cells treated with hMSC EVs from different groups: a control (i), unloaded EVs (ii), and EVs loaded via sonication (iii). (B) Live/dead assay images for the cells treated with hMSC EVs lyophilized with trehalose (i), EVs lyophilized without trehalose (ii), fresh EVs (iii), and a control (iv). Scale bar: 100 μm.

Table S1. Primer information for RT-qPCR analysis.

Primer Number	Gene Name	Primer Name in Database	Primer Sequence (5′-3′)
1	ACTB	Bactin F	GTACTCCGTGTGGATCGGCG
		Bactin R	AAGCATTTGCGGTGGACGATGG
2	GAPDH	GAPDH-F	TCACTGCCACCCAGAAGACTG
		GAPDH-R	GGATGACCTTGCCCACAGC
3	TNF-α	Forward	TGGCCAATGGCGTGGAGCTG
		Reverse	GTAGGAGACGGCGATGCGGC
4	IL-6	Forward	GAACTCCTTCTCCACAAGCG
		Reverse	TTTTCTGCCAGTGCCTCTTT
5	IL-12β	Forward	CCAAGGGGTGACGTGCGGAG

		Reverse	GGTGGGTCAGGTTTGATGATGTCCC
6	CD163	Forward	CCAGTCCCAAACACTGTCCT
		Reverse	ATGCCAGTGAGCTTCCCGTTCAGC
7	TGFβ	Forward	CCTACATTTGGAGCCTGGAC
		Reverse	TGTCCTTAAATACAGCCCCC
8	IL-10	Forward	AAGCCTGACCACGCTTTCTA
		Reverse	ATGAAGTGGTTGGGGAATGA
9	MMP2	MMP2 F1	CATCGCTCAGATCCGTGGTG
		MMP2 R1	GCATCAATCTTTTCCGGGAGC
10	MMP3	MMP3 F1	CCATCTCTTCCTTCAGGCGT
		MMP3 R1	ATGCCTCTTGGGTATCCAGC
11	MMP9	MMP9-F	GGCCACTACTGTGCCTTTGAG
		MMP9-R	AATCGCCAGTACTTCCCATCC
12	CLIC6	CLIC6-F1	GCAGTTTTACCCCAGCAGTCA
		CLIC6-R1	AATCTGCCCCCTAACCAGATG
13	PLEC	PLEC-F1	CTGTAGAGGGCCCTGGTGTTT
		PLEC-R1	GGGGCTTCTCTGGGTAGACTG
14	PLTP	PLTP-F1	CCCTGACTGAGAGGAACTGGA
		PLTP-R1	TCCATGGCAGAGTCGAAGAAG
15	TTR	TTR-F1	TGGGAAAACCAGTGAGTCTGG
		TTR-R1	GATGCCAAGTGCCTTCCAGTA
16	IGFBP7	IGFBP7-F1	GGAACAAGGTAAAAAGGGGTCA
		IGFBP7-R1	AGCACCCAGCCAGTTACTTCA
17	MSX1	MSX1-F1	GCCTTCCCTTTAACCCTCACA
		MSX1-R1	GGGACTCTTCCAGCCACTTTT
18	DCN	DCN-F1	GATTTGGTGGGGAGGCACTAT
		DCN-R1	GCCTCCTTTATGCCAACCTGT
19	LUM	LUM-F1	GGCAGGCCTATTTCATCACAA
		LUM-R1	AAGGTTTTGCACATCATTTGACAG
20	DLK	DLK1-F1	CTGAGAGTGTCGGGATCCTTG
		DLK1-R1	TGGATCGCTGTCTTTGAGCTT
21	AQP1	AQP1-F1	CAGCCCAAGGACAGTTCAGAG
		AQP1-R1	TCATGGCTAAGTGCACAGTGG
22	SMPD2-1	SMPD2-F1	GCCTGGGAGACTTTCTGAACC
		SMPD2-R1	AAGTGGTGTGCAGCTGGGTAG
23	SRSF5(hrs)-1	SRSF5-F1	CTTCTCGGATCGAGGCTTCTT
		SRSF5-R1	TCGAATCAACTGCGCTCATTA
24	SMPD3-1	SMPD3-F1	TTAAGAGACTCCAGGGCTGCTC
		SMPD3-R1	CGGGGATTGTCAAAAACAGTC
25	STAM1	STAM1-F1	CACTGGATTTTTGGGTTGCTC
		STAM1-R1	GTGGAAAACATTTTTCGCATGA
26	PDCD61P (ALIX)	PDCD61P F	TAAGTGCATCTGAGGGCCAAA
		PDCD61P R	GGGGCCTCCTTTCCTAGTTTC
27	PDCD61PI4 (ALIXI4)	PDCD61Pi4 F	TTGGCTAATCAGGCTGCAGAT
		PDCD61Pi4 R	TCACATGCAAAGTAAGCAAGTGTT
28	STAM2	STAM2-F1	GAGGCATTATGTTGCTTGTGTGA
		STAM2-R1	GGTGAGGGGTGGCTACTGTTA
29	RAB27B	RAB27B F	TCCATGAAGCTGCTTGTCTCA
		RAB27B R	GTTGGGTCTCCACCCAGAAAT
30	RAB7A	RAB7A F	TGCTCCCTTCCTAGGATCTGC
		RAB7A R	CAGAAGAACTCAGCCCACACC

31	RAB27A	RAB27A F	GCATGTTTCAGTTTTCAAGAACCA
		RAB27A R	AAAGGTGGCTTTTGTGTGTGC

Table S2. A list of antibodies.

Primary Antibody	Origin/ Isotype	Supplier/Cat#	Dilution
CD63	Mouse IgG_1	Santa Cruz, SC-5275	1:1000
HSC70	Rabbit IgG	Cell Signaling Technology, 8444	Western blot: 1:1000
TSG101	Rabbit IgG	ProSci (VWR, 10107-340)	Western blot: 1:1000
Syntenin-1	Mouse IgG1	SANTA, CRUZ/sc-100336	Western blot: 1:1000
Calnexin (Negative exosome marker)	Rabbit IgG	GeneTex, GTX101676	Western blot: 1:1000
**Secondary Antibody**	**Origin/ Isotype**	**Supplier/ Cat#**	**Dilution**
IRDye® 800CW	Goat anti-mouse IgG1	LI-COR, 926-32350	Western blot: 1:5,000
IRDye® 800CW	Goat anti-Rabbit IgG (H + L)	LI-COR/ 926-32211	Western blot: 1:5,000

Table S3. Abbreviation list.

Abbreviation	Full Wording
ChP	Choroid Plexus
EV	Extracellular Vesicle
hiPSC	Human induced pluripotent stem cell
VWBR	Vertical-Wheel bioreactor
CSF	Cerebrospinal fluid
CNS	Central nervous system
BBB	Blood brain barrier
BCSFB	Blood-cerebrospinal fluid barrier
TNF	Tumor necrosis factor
hMSC	Human mesenchymal stem cells
ESCRT	Endosomal Sorting Complexes Required for Transport
OCT4	Octamer-binding transcription factor 4
SOX2	SRY-box transcription factor 2
Rho-kinase	ROCK
MEM	Minimal Essential Medium
FBS	Fetal Bovine Serum
EDTA	Trypsin/ethylenediaminetetraacetic acid
ULA	Ultra-low attachment
DMEM/F12	Dulbecco’s Modified Eagle Medium/Nutrient Mixture F-12
BMP	Bone morphogenetic protein
PEG	Polyethylene glycol
NTA	Nanoparticle Tracking Analysis
DLS	Dynamic light scattering
TEM	Transmission Electron Microscopy
RIPA	radio-immunoprecipitation assay
HFIP	hexafluor-2-propanole
SD	Standard Deviation
RT-qPCR	Quantitative reverse transcription polymerase chain reaction
TTR	Transthyretin
PLTP	phospholipid transfer protein
PLEC	Plectin
DCN	Decorin

LUM	Lumican
SLRP	Small leucine-rich proteoglycan
IGFBP7	Insulin-like growth-factor-binding protein 7
AQP1	Aquaporin 1
MSX1	Msh homeobox 1
ECM	Extracellular matrix
MMP	Matrix metalloproteinase
PVP40	Polyvinylpyrrolidone 40
